# Trastuzumab combined with doublet or single-agent chemotherapy as first-line therapy for HER2-positive metastatic breast cancer

**DOI:** 10.1007/s10549-017-4592-y

**Published:** 2017-11-29

**Authors:** Y. F. Yu, Y. Wang, T. P. Fu, K. Chen, J. Q. Liu, H. R. Yao

**Affiliations:** 1 Guangdong Provincial Key Laboratory of Malignant Tumor Epigenetics and Gene Regulation, Breast Tumor Center, Sun Yat-sen Memorial Hospital, Sun Yat-sen University, No. 33 Yinfeng Road, Haizhu District, Guangzhou, Guangdong 510288 People’s Republic of China; 2 Department of Surgery, State Key Laboratory of Oncology in South China, Collaborative Innovation Center for Cancer Medicine, Sun Yat-sen University Cancer Center, Guangzhou, Guangdong People’s Republic of China; 3 Department of Oncology, Sun Yat-sen Memorial Hospital, Sun Yat-sen University, Guangzhou, Guangdong People’s Republic of China

**Keywords:** Metastatic breast cancer, HER2-positive, Trastuzumab, Single-agent chemotherapy, Doublet-agent chemotherapy, Meta-analysis

## Abstract

**Purpose:**

To investigate the efficacy and safety of doublet versus single-agent chemotherapy (CT) plus trastuzumab (H) as first-line therapy for human epidermal growth factor 2 receptor (HER2)-positive metastatic breast cancer (MBC).

**Methods:**

We searched for randomized clinical trials (RCTs) that evaluated the treatment effects of single-agent or doublet CT+H as first-line therapies for HER2-positive MBC. The main outcomes measured for this study included the overall response rate (ORR), progression-free survival (PFS), and overall survival (OS). A meta-analysis and trial sequential analysis (TSA) were performed, and the study quality was evaluated using the GRADE framework. The PROSPERO registry number of our analysis is CRD42016043766.

**Results:**

The results from four RCTs including 1044 participants were pooled. Moderate-quality evidence indicated that compared with single-agent CT+H, doublet CT+H correlated better with prolonged PFS (hazard ratio [HR] 0.69, 95% confidence interval [CI] 0.63–0.75, *P* < 0.0001) and OS (HR = 0.90, 95% CI 0.88–0.92, *P* < 0.0001). However, moderate-quality evidence revealed no significant difference between the two regimens regarding the ORR (relative risk [RR] = 1.07, 95% CI 0.98–1.17, *P* = 0.157), which was confirmed by TSA, indicating that the cumulative Z-curve entered the futility area. Moderate-quality evidence indicated that treatment-related grade 3 or 4 toxicities of thrombocytopenia (RR = 4.08, *P* = 0.000), nausea/vomiting (RR = 4.26, *P* = 0.002), diarrhea (RR = 2.81, *P* = 0.002), and stomatitis (RR = 5.02, *P* = 0.003) were observed more frequently with doublet CT+H than with single-agent CT+H.

**Conclusions:**

Compared with single-agent CT, the combination of doublet CT with trastuzumab as first-line therapy for HER2-positive MBC is associated with longer PFS and OS, but more treatment-related grade 3 or 4 toxicities. Therefore, doublet CT appears to be an appropriate regimen for HER2-positive MBC with a good performance status.

**Electronic supplementary material:**

The online version of this article (10.1007/s10549-017-4592-y) contains supplementary material, which is available to authorized users.

## Introduction

Human epidermal growth factor receptor 2 (HER2), a transmembrane receptor and member of the tyrosine kinase receptor family, mediates tumor invasion, progression, and metastasis [1, 2]. HER2 protein overexpression or gene amplification occurs in approximately 20–25% of patients with metastatic breast cancer (MBC), and is characterized by aggressive tumor growth, a high possibility of recurrence, and decreased survival. Fortunately, the introduction of anti-HER2 therapy has alleviated many of these concerns [3, 4].

Blocking HER2 using antibodies with a high affinity to the extracellular domain of the receptor leads to antibody-dependent cell-mediated cytotoxicity (ADCC) and prevents the intracellular kinase domain of the receptor from engaging in signal transduction. This blockade results in inhibition of numerous mitogenic pathways in HER2-overexpressing malignant tumor cells [5, 6]. Trastuzumab (Herceptin [H], F. Hoffmann-La Roche Ltd, Basel, Switzerland) is a recombinant, humanized monoclonal antibody that inhibits HER2 signaling, and has been approved by the Food and Drug Administration (FDA) for treatment of HER2-positive MBC [7, 8]. Previous clinical studies have extensively evaluated the use of trastuzumab in MBC, and the results have demonstrated that it significantly increases the survival of patients with HER2-positive MBC. Additionally, a number of studies have shown that trastuzumab inhibits tumor growth, produces a high response rate, and improves patient prognosis when used in combination with chemotherapy (CT) [9, 10]. Trastuzumab exerts greater activity when used in combination due to enhancement in the blockade of HER2 signaling; thus, the combination of trastuzumab with standard taxane- and platinum-based CT is recommended. These combination therapies provide significant survival benefits to women with HER2-positive MBC [10–12].

The current National Comprehensive Cancer Network (NCCN) guidelines recommend trastuzumab combined with CT as first-line therapy for patients with HER2-positive MBC; however, whether doublet-agent CT is superior to single-agent CT when combined with trastuzumab remains unknown [13]. Moreover, other relevant guidelines regarding these treatment regimens are lacking. In a randomized phase III trial [14] that examined patients with HER2-positive MBC, the addition of carboplatin to paclitaxel and trastuzumab was associated with a higher overall response rate (ORR) and increased overall survival (OS) compared with those obtained with paclitaxel combined with trastuzumab. In contrast, the BCIRG 007 study [15] indicated that in women with HER2-positive MBC, the addition of carboplatin to docetaxel and trastuzumab did not significantly affect the ORR or OS compared with those obtained with docetaxel plus trastuzumab. To resolve such inconsistencies, a comprehensive, high-quality assessment of the most recent randomized clinical trials (RCTs) examining treatment strategies for patients with HER2-positive MBC is warranted. Thus, the current meta-analysis sought to assess the currently available evidence regarding the effectiveness of single-agent versus doublet CT when combined with trastuzumab-targeted therapy as first-line therapy for women with HER2-positive MBC.

## Methods

This study was registered with PROSPERO (registration number CRD42016043766). The recommendations in the *Cochrane Handbook for Systematic Reviews of Interventions* and the guidelines in the PRISMA statement were utilized to design, analyze, and report this meta-analysis [16, 17].

### Database search and trial selection

A systematic literature search of the PubMed, EMBASE, and Cochrane Central Register of Controlled Trials databases was performed to identify relevant RCTs published prior to July 2016. The population, intervention, comparison, and outcome (PICO) strategy was used with the following search terms: “trastuzumab,” “metastatic breast cancer,” “HER2 positive,” and “randomized clinical trial.” No restrictions were imposed regarding sample size, population, language, publication year, publication type, or publication status. The following criteria were applied: RCTs that compared the efficacy of H combined with “standard CT” (single-agent or doublet) for patients with HER2-positive MBC and original full-text articles that reported one or more of the following outcomes: ORR, disease control rate (DCR), progression-free survival (PFS), OS, and safety.

### Data extraction

The following baseline characteristics and outcomes were extracted: trial name (including first author, year of publication, and registry numbers for clinical trials), study design, treatment regimen, recruitment period, number of participants, participant and tumor characteristics, follow-up duration, median response duration, median OS, median PFS, and primary and secondary endpoints.

### Statistical analyses

All efficacy endpoints were subjected to intent-to-treat (ITT) analysis when possible. Dichotomous data were analyzed according to the relative risk (RR) and risk difference (RD), with the number of patients needed to treat to benefit (NNTB) and the number of patients needed to treat to harm (NNTH) represented by 1/RD. The DerSimonian and Laird random effects model [18] was utilized when *I* ^2^ > 50%; otherwise, the Mantel–Haenszel fixed effects model [19] was applied. For time-to-event data, estimated hazard ratios (HRs) were pooled using the inverse-variance method [20]. The median survival was summarized as the median ratio (MR). The 95% confidence interval (CI) was reported for all estimates. *P* < 0.05 was considered statistically significant. Potential publication bias was visually evaluated using funnel plots and the Copas selection model [21] and quantified using Begg’s [22] and Egger’s [23] unweighted regression tests. *P* < 0.05 indicated a publication bias. All *P* values were two-sided. Meta-analysis and trial sequential analysis (TSA) were conducted (Supplementary trial sequential analysis, available online). The evidence quality was evaluated using the GRADE framework (Supplementary evidence quality, available online). To ensure the reliability and accuracy of the results, two authors independently uploaded the data. Statistical analyses were performed using R version 3.3.2 (R Foundation for Statistical Computing, Vienna, Austria).

## Results

### Search strategy, results, and study characteristics

Altogether, 4575 potential studies were identified using the search criteria. We qualitatively examined each article, which resulted in the selection of four RCTs [14, 15, 24, 25] for inclusion in our meta-analysis (Supplementary Fig. S1, available online). The included trials and patient characteristics are presented in Table 1. The four RCTs [14, 15, 24, 25] were published between 2006 and 2014 by Robert et al. [14], Wardley et al. (NCT01038466) [24], Valero et al. (NCT00047255) [15], and Baselga et al. (NCT00294996) [25]. In total, 1044 participants were included (median age [range] 52 years [18–83]), with 196–363 participants included per study. Three of the four eligible studies were multicenter and/or international randomized trials that recruited participants from 1998 to 2009. Of the included trials, two trials [14, 15] examined the combination of H, taxanes (paclitaxel/docetaxel) and carboplatin; one trial [24] examined the combination of H, a taxane (docetaxel) and capecitabine; and one trial [25] examined the combination of a taxane (paclitaxel), an anthracycline (non-pegylated liposomal doxorubicin) and H (Supplementary Table S1, available online). The baseline patient and tumor characteristics, including patient performance status, disease involvement, clinicopathological tumor features, and prior therapy regimens, showed similar distributions between the study groups. The data revealed that nearly all (99%) of the patients had a pretreatment performance status of at least 80% or less than 2, based on the Karnofsky performance score (KPS) or the Eastern Cooperative Oncology Group performance status (ECOG-PS) score, respectively. All trials were determined to have an unclear or high risk of bias due to insufficient participants and the lack of personnel blinding (Supplementary Figs. S2 and S3, available online).

**Table 1:** Characteristics of the included randomized clinical trials

Characteristics	Robert et al. [14]		Wardley et al. [24] CHAT study		Valero et al. [15] BCIRG 007		Baselga et al. [25]	
Clinicaltrials.gov, number	NS		NCT01038466		NCT00047255		NCT00294996	
Study design	RCT, Phase III		RCT, Phase II		RCT, Phase III		RCT, Phase III	
Recruitment period	1998–2002		2002–2005		2001–2004		2006–2009	
No. of countries	2		NS		13		12	
No. of centers	83		43		80		83	
Regimen	HPC	HP	HTX	HT	HTC	HT	HPM	HP
No. of participants	98	98	112	110	132	131	181	182
Age (years) Median (range)	55 (35–81)	56 (33-83)	53 (24-82)	52 (23-78)	51 (18-75)	52 (18-75)	52 (22-79)	53 (30-76)
ECOG-PS or KPS, No. (%)								
0 or 100	59 (60.2)	60 (61.2)	112 (100)	110 (100)	132 (100)	131 (100)	113 (62.4)	112 (61.5)
1 or 80–90	35 (35.7)	35 (35.7)	0 (0)	0 (0)	0 (0)	0 (0)	68 (37.6)	70 (38.5)
2 or < 80	4 (4.1)	3 (3.1)	0 (0)	0 (0)	0 (0)	0 (0)	0 (0)	0 (0)
HER2 status, No. (%)								
IHC 3+/FISH+	66 (68.4)	64 (65.3)	104 (92.9)	103 (93.6)	132 (100)	131 (100)	177 (97.8)	179 (98.9)
IHC 2+	32 (31.6)	33 (34.7)	8 (7.1)	7 (6.4)	0 (0)	0 (0)	4 (2.2)	3 (1.1)
Hormonal receptor status, No. (%)								
ER+	51 (52)	63 (64.3)	50 (44.6)	39 (35.5)	NS	NS	NS	NS
PgR+	40 (40.8)	47 (48.0)	38 (33.9)	31 (28.2)	NS	NS	NS	NS
ER+/PgR+	NS	NS	56 (50.0)	45 (40.9)	86 (62.5)	95 (72.5)	75 (41.4)	81 (44.5)
Disease involvement, No. (%)								
Visceral	52 (53.1)	39 (39.8)	NS	NS	77 (58.3)	87 (66.4)	NS	NS
Lung	NS	NS	26 (23.2)	31 (28.2)	NS	NS	90 (49.7)	90 (49.5)
Bone	42 (42.9)	37 (37.7)	26 (23.2)	28 (25.5)	44 (33.3)	55 (41.9)	64 (35.4)	71 (39.0)
Liver	34 (34.7)	42 (429)	15 (13.4)	22 (20.0)	65 (49.2)	67 (51.1)	70 (38.7)	80 (44.0)
Soft tissue	46 (46.9)	52 (53.1)	32 (28.6)	42 (38.2)	NS	NS	NS	NS
Other^a^	9 (9.2)	3 (3.1)	9 (8.0)	10 (9.1)	NS	NS	113 (62.4)	108 (59.3)
Prior therapy, No. (%)								
Surgery	78 (79.6)	74 (75.5)	NS	NS	NS	NS	NS	NS
Chemotherapy	48 (49.0)	45 (45.9)	55 (49.1)	55 (50.0)	73 (55.7)	71 (53.8)	NS	NS
Radiotherapy	37 (37.8)	41 (41.8)	49 (43.8)	52 (47.3)	NS	NS	NS	NS
Hormonal therapy	39 (40.8)	50 (51.0)	35 (31.3)	36 (32.7)	48 (36.4)	35 (26.7)	NS	NS
Anthracycline	NS	NS	49 (43.8)	49 (44.5)	43 (32.6)	43 (32.8)	59 (32.6)	60 (33.0)
Taxane	NS	NS	NS	NS	12 (9.1)	14 (10.7)	14 (7.7)	12 (6.6)
Trastuzumab	NS	NS	NS	NS	NS	NS	2 (1.1)	4 (1.1)
No prior chemotherapy	NS	NS	NS	NS	59 (44.7)	57 (43.5)	NS	NS
Outcomes								
Follow-up (months)	52		26 (median)		84		44 (median)	
Median response duration (months)	13	11	15.9	13.4	10.7	9.4	18.1	15.3
Median OS (months)	35.7	32.2	46.0	40.2	37.4	37.1	33.6	29.0
Median PFS (months)	10.7	7.1	17.9	12.8	NR	NR	16.1	14.5
Primary and secondary end points	PFS, OS, ORR, DCR, Safety		PFS, OS, ORR, DCR, Safety		OS, ORR, DCR, Safety		PFS, OS, ORR, DCR, Safety	

*RCT* randomized clinical trial, *ECOG-PS* Eastern Cooperative Oncology Group performance status, *KPS* Karnofsky performance status, *IHC* immunohistochemistry, *FISH* fluorescence in situ hybridization, *HER2* human epidermal growth factor receptor 2, *ER* estrogen receptor, *PgR* progesterone receptor, *NS* not specified, *HPC* trastuzumab, paclitaxel and carboplatin, *HP* trastuzumab and paclitaxel, *HTX* trastuzumab, docetaxel and capecitabine, *HT* trastuzumab and docetaxel, *HTC* trastuzumab, docetaxel and carboplatin, *HT* trastuzumab and docetaxel, *HPM* trastuzumab, paclitaxel and non-pegylated liposomal doxorubicin, *HP* trastuzumab and paclitaxel, *DOR* duration of response, *PFS* progression-free survival, *OS* overall survival, *ORR* objective response rate, *DCR* disease control rate

^*^Other sites for metastatic disease included the heart, lymph nodes, adrenal glands, kidneys, and chest wall

### Efficacy

#### ORR did not significantly differ between the groups

**Fig. 1:**
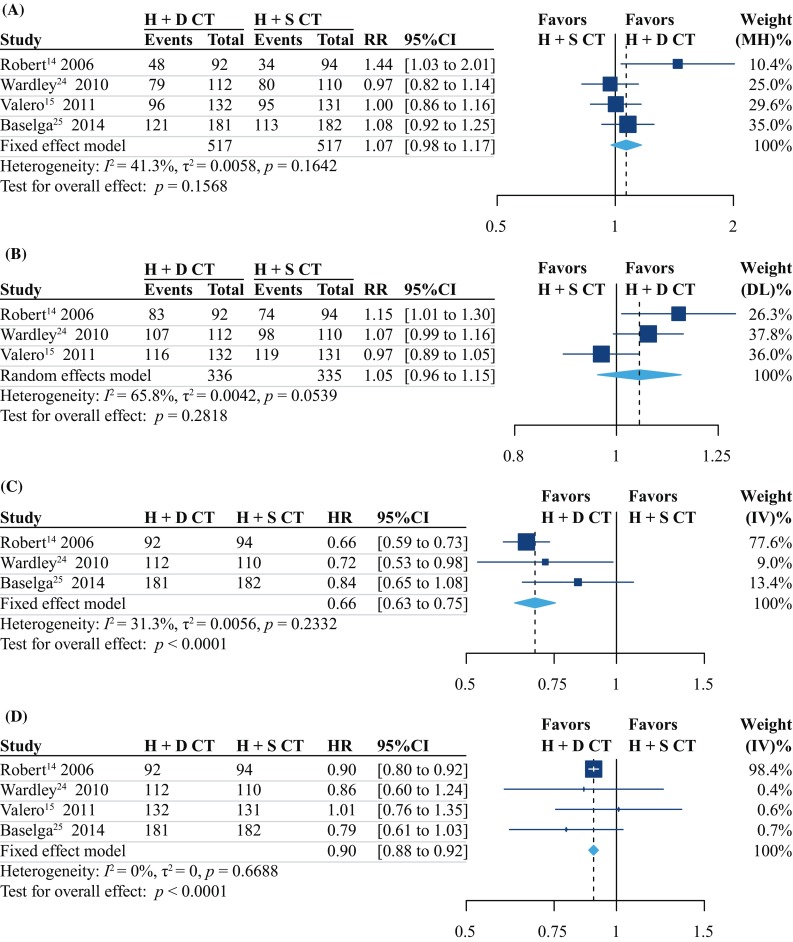
Forest plot of the **a** overall response rate, **b** disease control rate, **c** progression-free survival, and **d** overall survival for the two treatment groups. *RR* risk ratio; *HR* hazard ratio; *95% CI* 95% confidence interval; *H* trastuzumab; *CT* chemotherapy; *D* doublet; *S* single-agent

**Fig. 2:**
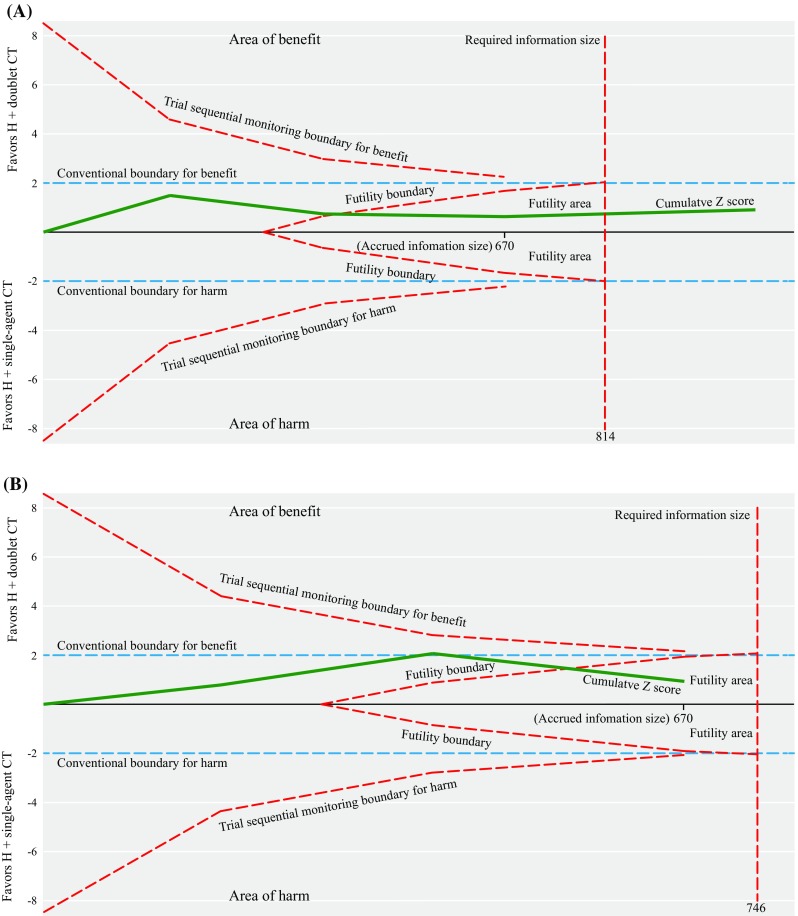
Trial sequential analysis results for the **a** overall response rate and **b** disease control rate for the two treatment groups. The diversity required information size of 814 (and adjacent trial sequential alpha spending monitoring boundaries) for the objective response rate was calculated based on an *α* value of 5% (two-sided), a power of 80%, an anticipated relative risk reduction of 20%, and an event proportion of 62.68% in the control arm, as estimated using a random effects model. The diversity required information size of 746 (and adjacent trial sequential alpha spending monitoring boundaries) for the disease control rate was calculated based on an α value of 5% (two-sided), a power of 80%, an anticipated relative risk reduction of 20%, and an event proportion of 86.86% in the control arm, as estimated using a random effects model. The solid green cumulative *Z* curves indicate the cumulative *Z* score from the inverse-variance model *Z* statistic when a new trial is added. The solid green cumulative *Z* curves cross the dashed red futility boundary, and trial sequential alpha spending monitoring boundaries represent the objective response rate and disease control rate. The horizontal dotted blue lines illustrate the traditional level of statistical significance (*P* = 0.05). *H* trastuzumab; *CT* chemotherapy

Four trials comprising 1034 total participants were included in the current meta-analysis to estimate the ORR. The pooled ORRs were 66 and 61% for doublet CT+H and single-agent CT+H, respectively. These results provided moderate-quality evidence indicating that no significant difference existed between the two groups in terms of the ORR (RR = 1.07, 95% CI 0.98–1.17, *P* = 0.157, *I* ^2^ = 41.3%; RD = 4%, 95% CI − 2 to 10%; Fig. 1a, Table 2). The results of Egger’s test (*P* = 0.955), Begg’s test (*P* = 1.000), and the Copas selection model indicated that there was no evidence of publication bias (Supplementary Fig. S4A, available online). We also subjected the ORR results to TSA, which provided sufficient and conclusive evidence indicating that no significant difference existed between the groups, and thus, further trials were not required (Fig. 2a).

**Table 2:** Efficacies of the two treatments

Outcomes	No. of participants	Relative effect			Risk difference	GRADE	
		Ratio (95% CI)	*P* value	*I* ^2^	(95% CI)	Quality	Importance
Complete response	671 (14, 15, 24)	RR 1.28 (0.90 to 1.82)	0.176	37.5%	4% (− 2 to 9%)	⊕⊕ΟΟ Low^a,b^	Important
Partial response	671 (14, 15, 24)	RR 1.07 (0.83 to 1.38)	0.601	61.9%	3% (− 9 to 16%)	⊕⊕ΟΟ Low^a,b^	Important
Overall response	1034 (14, 15, 24, 25)	RR 1.07 (0.98 to 1.17)	0.157	41.3%	4% (− 2 to 10%)	⊕⊕⊕Ο Moderate^a^	Critical
Stable disease	671 (14, 15, 24)	RR 1.02 (0.78 to 1.32)	0.910	40.0%	0.4% (− 6 to 7%)	⊕⊕ΟΟ Low^a,b^	Important
Disease control	671 (14, 15, 24)	RR 1.05 (0.96 to 1.15)	0.282	65.8%	5% (− 4 to 13%)	⊕⊕ΟΟ Low^a,c^	Important
Progressive disease	671 (14, 15, 24)	RR 0.59 (0.34 to 1.04)	0.066	22.1%	− 5% (− 11 to 1%)	⊕⊕ΟΟ Low^a,b^	Important
Median duration of response	1034 (14, 15, 24, 25)	MR 1.17 (1.10 to 1.25)	< 0.0001	0.0%	–	⊕⊕⊕Ο Moderate^a^	Important
Progression-free survival	771 (14, 24, 25)	HR 0.69 (0.63 to 0.75)	< 0.0001	31.3%	–	⊕⊕⊕Ο Moderate^a^	Critical
Median progression-free survival	771 (14, 24, 25)	MR 1.32 (1.09 to 1.60)	0.004	85.7%	–	⊕⊕ΟΟ Low^a,c^	Important
Overall survival	1034 (14, 15, 24, 25)	HR 0.90 (0.88 to 0.92)	< 0.0001	0.0%	–	⊕⊕⊕Ο Moderate^a^	Critical
Median overall survival	1034 (14, 15, 24, 25)	MR 1.11 (1.04 to 1.18)	0.001	9.1%	–	⊕⊕⊕Ο Moderate^a^	Important

*CI* confidence interval; *HR* hazard ratio; *RR* risk ratio; *MR* median ratio

Grade Working Group grades of evidence

⊕⊕⊕⊕High quality: further research is very unlikely to change our confidence in the estimated effect

⊕⊕⊕ΟModerate quality: further research is likely to have an important impact on our confidence in the estimated effect and might change the estimate

⊕⊕ΟΟLow quality: further research is very likely to have an important impact on our confidence in the estimated effect and might change the estimate

⊕ΟΟΟVery low quality: we are very uncertain about the estimate

^a^Downgraded (− 1) for risk of bias: all trials were judged as having an unclear or high risk of bias related to the blinding of participants and personnel

^b^Downgraded (− 1) for imprecision: small sample bias might exist, or the 95% confidence intervals are wide; the study includes no effect and fails to exclude important benefits or serious harmful effects

^c^Downgraded (− 1) for inconsistency: substantial heterogeneity (*I* ^2^ > 50%) was found among the trials

The median response duration was reported in four trials (1034 participants). The median response of the doublet CT+H group ranged from 10.7 to 18.1 months, whereas the median response of the single-agent CT+H group ranged from 9.4 to 15.3 months. The pooled results provided moderate-quality evidence indicating that the median response duration was significantly longer in the doublet CT+H group than in the single-agent CT+H group (MR = 1.17, 95% CI 1.10–1.25, *P* < 0.0001, *I* ^2^ = 0.0%; Table 2; Supplementary Fig. S5A, available online).

#### The DCR was not significantly different between the groups

DCR data were available for three RCTs [14, 15, 24] (671 participants). The pooled DCRs for the doublet CT+H and single-agent CT+H groups were 92 and 87%, respectively. The pooled results provided low-quality evidence indicating that no significant difference in the DCR existed between the groups (RR = 1.05, 95% CI 0.96 to 1.15, *P* = 0.282, *I* ^2^ = 65.8%; RD = 5%, 95% CI − 4 to 13%; Fig. 1b, Table 2). No significant publication bias was identified based on the results of Egger’s test (*P* = 0.541), Begg’s test (*P* = 1.000), and the Copas selection model (Supplementary Fig. S4B, available online). We also subjected the DCR results to TSA, which indicated that additional trials were not required and were unlikely to alter the outcomes (Fig. 2b).

#### Doublet CT+H was associated with longer PFS

We performed a pooled analysis of the three trials [14, 24, 25] (771 participants) that reported sufficient PFS data. The median PFS values for the doublet CT+H and single-agent CT+H groups ranged from 10.7 to 17.9 and from 7.1 to 14.5 months, respectively. The trials provided low-quality evidence indicating that the doublet CT+H group had a significantly longer PFS than did the single-agent CT+H group (MR = 1.32, 95% CI 1.09–1.60, *P* = 0.004, *I* ^2^ = 85.7%; Supplementary Fig. S5B, available online). Overall, the results of our meta-analysis provide moderate-quality evidence that doublet CT+H is associated with a 31% reduction in disease-progression risk compared with the disease-progression risk with single-agent CT+H (HR = 0.69, 95% CI 0.63–0.75, *P* < 0.0001, *I* ^2^ = 31.3%; Fig. 1c, Table 2). The results of Egger’s test (*P* = 0.396), Begg’s test (*P* = 1.000), and the Copas selection model (Supplementary Fig. S4C, available online) indicated that there was no evidence of publication bias regarding PFS.

#### Doublet CT+H was associated with longer OS

All four trials (1034 participants) reported comparable median OS values for the doublet CT+H and single-agent CT+H groups, ranging from 33.6 to 46.0 months and from 29.0 to 40.2 months, respectively. The pooled results provided moderate-quality evidence indicating that the doublet CT+H group had a significantly longer OS than the single-agent CT+H group (MR = 1.11, 95% CI 1.04–1.18, *P* = 0.001, *I* ^2^ = 9.1%; Supplementary Fig. S5C, available online). Overall, the meta-analysis provided moderate-quality evidence showing that in women with HER2-positive MBC, doublet CT+H is associated with a 10% reduction in the risk of death compared with that with single-agent CT+H (HR = 0.90, 95% CI 0.88–0.92, *P* < 0.0001, *I* ^2^ = 0%; Fig. 1D, Table 2). The results of Egger’s test (*P* = 0.814), Begg’s test (*P* = 1.000), and the Copas selection model (Supplementary Fig. S4D, available online) indicated that there was no evidence of publication bias regarding OS.

### Safety

**Fig. 3:**
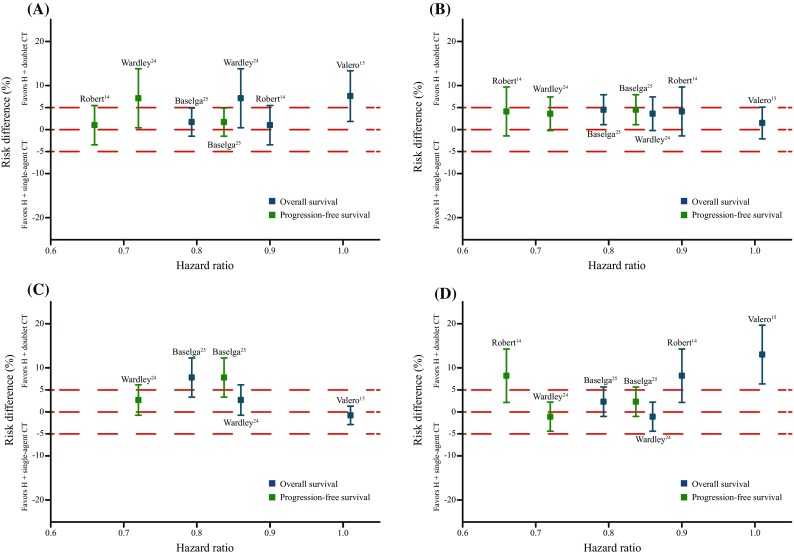
Effects of the two treatment approaches on absolute grade 3 or 4 toxicities for **a** diarrhea, **b** nausea/vomiting, **c** stomatitis, and **d** thrombocytopenia

For non-hematologic toxicities, the meta-analysis provided moderate-quality evidence showing that compared with single-agent CT+H, doublet CT+H significantly increased the risk of nausea/vomiting (RR = 4.26, *P* = 0.002; NNTH = 25), diarrhea (RR = 2.81, *P* = 0.002; NNTH = 25), and stomatitis (RR = 5.02, *P* = 0.003; NNTH = 25). For hematologic toxicities, the meta-analysis provided moderate-quality evidence indicating that doublet CT+H significantly increased the risk of thrombocytopenia (RR = 4.08, *P* = 0.000; NNTH = 20). We also examined whether the potential benefit of doublet CT+H was offset by its higher toxicity rate by comparing the benefits (HR) with the risks of grade 3 or 4 thrombocytopenia, stomatitis, nausea/vomiting, and diarrhea (as percentages) between the doublet CT+H and single-agent CT+H groups (Fig. 3). There was only a slight association between the risk rates for these grade 3 or 4 toxicities and the doublet CT+H intervention. The safety results are summarized in Table S2 in the Supplemental Materials section (available online).

### Study withdrawals

Three trials [14, 24, 25] (781 participants) reported that 89 patients discontinued therapy as a result of treatment-related toxicities. The pooled RR provided very low-quality evidence that revealed no significant difference in the number of patients who discontinued therapy between the doublet CT+H and single-agent CT+H groups (10% vs. 8%, respectively; RR = 1.15, 95% CI 0.50–2.65, *P* = 0.747, *I* ^2^ = 53.9%; RD = 2%, 95% CI − 5 to 8%; Supplementary Table S2 and Supplementary Fig. S6A, available online). No publication bias was identified from the results of Egger’s test (*P* = 0.872), Begg’s test (*P* = 1.000), or the Copas selection model (Supplementary Fig. S4E, available online).

Three trials [15, 24, 25] (848 participants) reported 27 deaths during the drug therapy period. The pooled incidence of mortality (3%) was the same for both treatment arms. The pooled RR provided very low-quality evidence showing the absence of a significant difference in mortality between the doublet CT+H and single-agent CT+H groups (RR = 0.80, 95% CI 0.38–1.68, *P* = 0.557, *I* ^2^ = 0.0%; RD = − 1%, 95% CI − 3 to 2%; Supplementary Table S2 and Supplementary Fig. S6B, available online). No publication bias was identified from the results of Egger’s test (*P* = 0.993), Begg’s test (*P* = 1.000), or the Copas selection model (Supplementary Fig. S6F, available online).

## Discussion

### Principal findings

To the best of our knowledge, this study constitutes the first meta-analysis to comprehensively and systematically evaluate the efficacy and safety of single-agent CT+H versus doublet CT+H as first-line therapy for patients with HER2-positive MBC. Our investigation describes the effects of the duration and efficacy of these treatments on survival. Overall, 1044 patients were included, and no significant differences in the ORR (66% vs. 61%, *P* = 0.157) or DCR (92% vs. 87%, *P* = 0.282) were found between the groups. PFS (*P* < 0.0001) and OS (*P* < 0.0001) were significantly longer in the doublet CT+H group than in the single-agent CT+H group, and the results showed a clinically meaningful difference in median survival. The results of safety analyses showed that both groups tolerated the drug regimens well. Fewer grade 3 or 4 adverse events were associated with single-agent CT+H than with doublet CT+H.

### Implications for clinical practice

The current NCCN guidelines recommend pertuzumab plus trastuzumab in combination with a taxane as the preferred first-line therapy for HER2-positive MBC [13, 26]. However, pertuzumab has not yet been approved as a treatment for patients with HER2-positive MBC in several countries, including China. Therefore, first-line trastuzumab in combination with a selected CT regimen (e.g., paclitaxel ± carboplatin, docetaxel, vinorelbine, and capecitabine) is another therapeutic option for HER2-positive MBC [13]. However, whether doublet CT is superior to single-agent CT in combination with trastuzumab for such patients has not been clarified. The results of a prior literature-based meta-analysis suggested that combination CT results in a better ORR than a sequential single-agent CT in patients with HER2-negative MBC. However, no difference in OS or PFS was found when these treatment strategies were compared in HER2-positive MBC [27]. These findings support international guidelines that recommend the use of sequential monotherapy for most cases of MBC, unless rapid disease progression, or life-threatening visceral metastases occurs, or the need for rapid symptom or disease control is present, in which case combination CT is preferred [28]. Nonetheless, whether the most effective chemotherapy regimen should consist of single or doublet chemotherapeutic agents combined with trastuzumab for HER2-positive disease remains unclear.

In the present meta-analysis, although no statistically significant difference in the ORR was found between the groups, doublet CT+H resulted in a significantly longer median response duration than single-agent CT+H (MR = 1.17, *P* < 0.0001). The PFS and OS were also significantly longer in the doublet CT+H group than in the single-agent CT+H group. Altogether, these findings indicate that greater inconsistency and fewer direct correlations may exist between the response rate and survival benefits.

One explanation for the differences in PFS and OS but not the ORR between the study arms is that doublet CT+H resulted in a prolonged median response duration, which might also underlie the inconsistencies noted between the short-term response and long-term survival benefits. This phenomenon was also observed in other trials. For instance, in the FIRE-3 trial, no significant difference was identified in the ORR of patients with metastatic colorectal cancer when either cetuximab or bevacizumab was added to fluorouracil with folinic acid and irinotecan (FOLFIRI). However, cetuximab was associated with a longer OS, which suggests that FOLFIRI plus cetuximab should be the preferred first-line regimen for these patients [29]. The inconsistencies noted in the abovementioned study might be due to the deepness of response (DpR) to therapy, as the DpR might not have been adequately captured using RECIST guidelines when comparing the different targeted therapies.

Another reason that a significant difference in long-term survival benefit was observed but no apparent difference in the short-term response was found between the groups could be the immunologic effect of the treatment. The mechanisms of action of trastuzumab include the direct arrest of cell growth, the induction of apoptosis, the inhibition of HER2 shedding, and the recruitment of immune effector cells that mediate tumor cell lysis. The recruitment of immune effector cells and subsequent cell lysis, designated ADCC, depends on the expression of Fc receptors (FcRs) in innate immune cells [30]. A recent study conducted using FinHER adjuvant samples [31] was the first to demonstrate an association between high numbers of tumor-infiltrating lymphocytes and an increased benefit of trastuzumab in HER2+ MBC. Thus, although trastuzumab is thought to act primarily on tumor cells, antitumor immunity might also underlie the efficacy of anti-HER2 treatment.

Recent data have indicated that both nivolumab and pembrolizumab significantly improved OS but not PFS [32–35], which suggests that a divide exists between response-based endpoints and survival in some settings, even for programmed death 1 (PD-1) inhibitors. Immunotherapeutic agents produce antitumor effects by inducing cancer-specific immune responses or modifying native immune processes. The resulting clinical response patterns extend beyond those of cytotoxic agents and can manifest after an initial increase in the tumor burden or the appearance of new lesions (i.e., disease progression).

Therefore, RECIST or WHO criteria, which are designed to detect early effects of cytotoxic agents, might not provide a complete assessment of immunotherapeutic agents. The modification of these criteria to capture the unique response patterns generated by immunotherapeutic agents has previously been proposed and is becoming increasingly recognized as necessary for the proper evaluation of these agents [36]. In a case study of an ipilimumab-treated patient who exhibited disease progression at a 12-week tumor assessment, histologic analyses indicated that the increase in lesion size likely resulted from T cell infiltration as opposed to tumor cell proliferation [37]. Thus, inflammation in baseline lesions might be misinterpreted as disease progression (a version of the “tumor flare reaction”). Therefore, for immunotherapeutic agents that induce tumor shrinkage in some patients, the evaluation of immune-related criteria will likely provide a more comprehensive assessment of clinical activity and might help explain why patients with apparent progressive disease experience better long-term survival in some cases.

Improving survival of patients with metastatic disease is a worthy goal, even if objective responses are not improved. Furthermore, evaluating the statistical and clinical significance of intervention outcomes [38] by assessing the magnitudes of effect sizes, patient-reported minimum relevant differences and self-perceived meaningful benefits [39], provides considerable insight into treatment-related toxicity, including grade 3 or 4 hematologic and non-hematologic toxicities, reasons for therapy discontinuation, and mortality during therapy. Moreover, patient performance status should be equally considered when reporting prognostic factors and associated outcomes. Previous studies have demonstrated that patients with a good pre-CT performance status may have a clinically significantly longer OS than expected [24]. The results of our toxicity analysis indicated that nearly all (99%) of the patients had a pretreatment performance status of at least 80% or less than 2, based on the KPS or the ECOG-PS score, respectively, and that treatment-related grade 3 or 4 hematologic and non-hematologic toxicities occurred more frequently with doublet CT+H than with single-agent CT+H. However, no significant differences in mortality or discontinued therapy due to treatment-related toxicities were found between the groups. Given that CT is a frontline therapy for HER2-positive MBC in a variety of clinical settings, determining the pretreatment performance status of a patient is essential. Overall, we believe that treatment regimens should be selected in the context of effectiveness, performance status, comorbidities, and toxicity profiles. Our findings support the use of doublet CT+H as first-line therapy for patients with HER2-positive disease unless a patient exhibits a poor pretreatment performance status, in which case single-agent CT+H will be recommended. Several factors limited our confidence in the effect estimates and CIs in our meta-analysis (Supplementary limitations, available online).

## Conclusion

In conclusion, the results of this meta-analysis indicate that compared with single-agent CT+H, doublet CT+H results in prolonged PFS and OS but more treatment-related toxicities when used as first-line therapy for patients with HER2-positive MBC. Moreover, compared with patients who received single-agent CT+H, those who received doublet CT+H showed a non-significant trend toward improved ORR. Based on our findings, we recommend doublet CT+H as first-line therapy for patients with good pretreatment performance status; however, in the case of patients with poor performance status, single-agent CT+H is recommended, regardless of the desire to rapidly reduce the tumor burden.

## Electronic supplementary material

Below is the link to the electronic supplementary material.

Supplementary material 1 (DOC 1719 kb)
