# Supplementary material for: Trastuzumab combined with doublet or single-agent chemotherapy as first-line therapy for HER2-positive metastatic breast cancer
Source: Breast Cancer Res Treat. 2017 Nov 29;168(2):337–48. doi: 10.1007/s10549-017-4592-y (PMC5838135; doi:10.1007/s10549-017-4592-y)
Supplement: Supplementary file 1 — Supplementary material 1 (DOC 1719 kb) [file 10549_2017_4592_MOESM1_ESM.doc]

**Supplementary Materials**

**Supplementary Methods.** Trial sequential analysis.

**Supplementary Methods**. Evidence quality.

**Supplementary Discussion.** Limitations.

**Supplementary Fig****. S1.** Flow chart of the selection of randomized controlled trials included in the meta-analysis.

**Supplementary Fig. S2.** Risk of bias summary. Green indicates a low risk of bias, yellow indicates an unclear risk of bias, and red indicates a high risk of bias.

**Supplementary Fig. S3.** Risk of bias graph. Green indicates a low risk of bias, yellow indicates an unclear risk of bias, and red indicates a high risk of bias.

**Supplementary Fig. S4.** Analysis of publication bias for the two treatment groups.

**Supplementary Fig. S5.** Forest plot of the (A) median duration of response, (B) median progression-free survival, and (C) median overall survival for the two treatment groups. MR, median ratio; 95% CI, 95% confidence interval; H, trastuzumab; CT, chemotherapy; D, doublet; S, single-agent.

**Supplementary Fig. S6.** Forest plot of the (A) discontinued therapy and (B) mortality for the two treatment groups. RR, risk ratio; HR, hazard ratio; 95% CI, 95% confidence interval; H, trastuzumab; CT, chemotherapy; D, doublet; S, single-agent.

**Supplementary Table S1.** Treatment summary for the two treatment groups.

**Supplementary Table S2.** Summary of the safety results for the two treatment regimens.

**Supplementary Methods**. Trial sequential analysis

The use of trial sequential analysis (TSA) in a meta-analysis enables the calculation of the required information size (i.e., number of participants), provides monitoring boundaries to decide whether a trial could be terminated early, and indicates whether a *P* value is sufficient to indicate a reliable effect for benefit, harm, or futility before the required information size is reached [1-5]. These analyses were conducted using TSA Beta (version 0.9) [6].

**Supplementary Methods**. Evidence quality.

Risk of bias was assessed using the components recommended by the Cochrane Collaboration [7]. Two authors (Y.F. Yu. and T.P. Fu.) independently used the GRADE system [8] to evaluate the overall quality of the evidence according to the risk of bias [9], inconsistency [10], indirectness [11], imprecision [12], and publication bias [13]. The quality of evidence was defined as very low, low, moderate, or high [14, 15]. The analyses were conducted with GRADE Profiler (GRADEpro, version 3.6) and Review Manager 5.3 (Review Manager 2014) in addition to using TSA (6). Discrepancies were resolved by a third party (H.R.Y.).

**Supplementary Discussion.** Limitations.

Despite the strengths of this meta-analysis and the discovery of novel insights into the prolonged PFS and OS obtained with doublet agent CT+H as first-line therapy compared with single-agent CT+H in HER2-positive MBC, our study has two main limitations. One limitation is that only a small number of trials were included in the safety analysis, resulting in the quality of this evidence being graded as low or very low. Additionally, only a limited number of trials evaluated the associations between clinicopathological characteristics and prognosis after systemic therapy in HER2-positive MBC patients. Therefore, additional well-designed studies are needed to confirm our findings.

**References**

1. Brok J, Thorlund K, Gluud C, Wetterslev J (2008) Trial sequential analysis reveals insufficient information size and potentially false positive results in many meta-analyses. J Clin Epidemiol 61:763–769. doi:10.1016/j.jclinepi.2007.10.007

2. Brok J, Thorlund K, Wetterslev J, Gluud C (2009) Apparently conclusive meta-analyses may be inconclusive–trial sequential analysis adjustment of random error risk due to repetitive testing of accumulating data in apparently conclusive neonatal meta-analyses. Int J Epidemiol 38:287–298. doi:10.1093/ije/dyn188

3. Wetterslev J, Thorlund K, Brok J, Gluud C (2008) Trial sequential analysis may establish when firm evidence is reached in cumulative meta-analysis. J Clin Epidemiol 61:64–75. doi:10.1016/j.jclinepi.2007.03.013

4. Thorlund K, Devereaux PJ, Wetterslev J, Guyatt G, Ioannidis JP, Thabane L, Gluud LL, Als-Nielsen B, Gluud C (2009) Can trial sequential monitoring boundaries reduce spurious inferences from meta-analyses? Int J Epidemiol 38:276–286. doi:10.1093/ije/dyn179

5. Thorlund K, Imberger G, Walsh M, Chu R, Gluud C, Wetterslev J, Guyatt G, Devereaux PJ, Thabane L (2011) The number of patients and events required to limit the risk of overestimation of intervention effects in meta-analysis–a simulation study. PLoS One 6:e25491. doi:10.1371/journal.pone.0025491

6. Thorlund K, Engstrøm J, Wetterslev J, Brok J, Imberger G, Gluud C. (2011) User manual for trial sequential analysis (TSA). Copenhagen Trial Unit, Centre for Clinical Intervention Research, Copenhagen, Denmark. p. 1-115. Available from www.ctu.dk/tsa.

7. Higgins JPT, Green S (2011) Cochrane handbook for systematic reviews of interventions, version 5.1.0. The Cochrane Collaboration. Retrieved from citeulike-article-id: 10329727.

8. Atkins D, Best D, Briss PA, Eccles M, Falck-Ytter Y, Flottorp S, Guyatt GH, Harbour RT, Haugh MC, Henry D, Hill S, Jaeschke R, Leng G, Liberati A, Magrini N, Mason J, Middleton P, Mrukowicz J, O’Connell D, Oxman AD, Phillips B, Schünemann HJ, Edejer T, Varonen H, Vist GE, Williams JW, Zaza S, GRADE Working Group (2004) Grading quality of evidence and strength of recommendations. BMJ 328:1490. doi:10.1136/bmj.328.7454.1490

9. Guyatt GH, Oxman AD, Vist G, Kunz R, Brozek J, Alonso-Coello P, Montori V, Akl EA, Djulbegovic B, Falck-Ytter Y, Norris SL, Williams JW, Atkins D, Meerpohl J, Schünemann HJ (2011) GRADE guidelines: 4. Rating the quality of evidence–study limitations (risk of bias). J Clin Epidemiol 64:407–415. doi:10.1016/j.jclinepi.2010.07.017

10. Guyatt GH, Oxman AD, Kunz R, Woodcock J, Brozek J, Helfand M, Alonso-Coello P, Glasziou P, Jaeschke R, Akl EA, Norris S, Vist G, Dahm P, Shukla VK, Higgins J, Falck-Ytter Y, Schünemann HJ, GRADE Working Group (2011) GRADE guidelines: 7. Rating the quality of evidence–inconsistency. J Clin Epidemiol 64:1294–1302. doi:10.1016/j.jclinepi.2011.03.017

11. Guyatt GH, Oxman AD, Kunz R, Woodcock J, Brozek J, Helfand M, Alonso-Coello P, Falck-Ytter Y, Jaeschke R, Vist G, Akl EA, Post PN, Norris S, Meerpohl J, Shukla VK, Nasser M, Schünemann HJ, GRADE Working Group (2011) GRADE guidelines: 8. Rating the quality of evidence–indirectness. J Clin Epidemiol 64:1303–1310. doi:10.1016/j.jclinepi.2011.04.014

12. Guyatt GH, Oxman AD, Kunz R, Brozek J, Alonso-Coello P, Rind D, Devereaux PJ, Montori VM, Freyschuss B, Vist G, Jaeschke R, Williams JW, Murad MH, Sinclair D, Falck-Ytter Y, Meerpohl J, Whittington C, Thorlund K, Andrews J, Schünemann HJ (2011) GRADE guidelines 6. Rating the quality of evidence--imprecision. J Clin Epidemiol 64:1283–1293. doi:10.1016/j.jclinepi.2011.01.012

13. Guyatt GH, Oxman AD, Montori V, Vist G, Kunz R, Brozek J, Alonso-Coello P, Djulbegovic B, Atkins D, Falck-Ytter Y, Williams JW, Meerpohl J, Norris SL, Akl EA, Schünemann HJ (2011) GRADE guidelines: 5. Rating the quality of evidence--publication bias. J Clin Epidemiol 64:1277–1282. doi:10.1016/j.jclinepi.2011.01.011

14. Guyatt GH, Oxman AD, Vist GE, Kunz R, Falck-Ytter Y, Alonso-Coello P, Schünemann HJ, GRADE Working Group (2008) GRADE: an emerging consensus on rating quality of evidence and strength of recommendations. BMJ 336:924–926. doi:10.1136/bmj.39489.470347.AD

15. Higgins JPT, Thompson SG, Deeks JJ, Altman DG (2003) Measuring inconsistency in meta-analyses. BMJ 327:557–560. doi:10.1136/bmj.327.7414.557

**Supplementary figures**

**Supplementary Fig. S1**


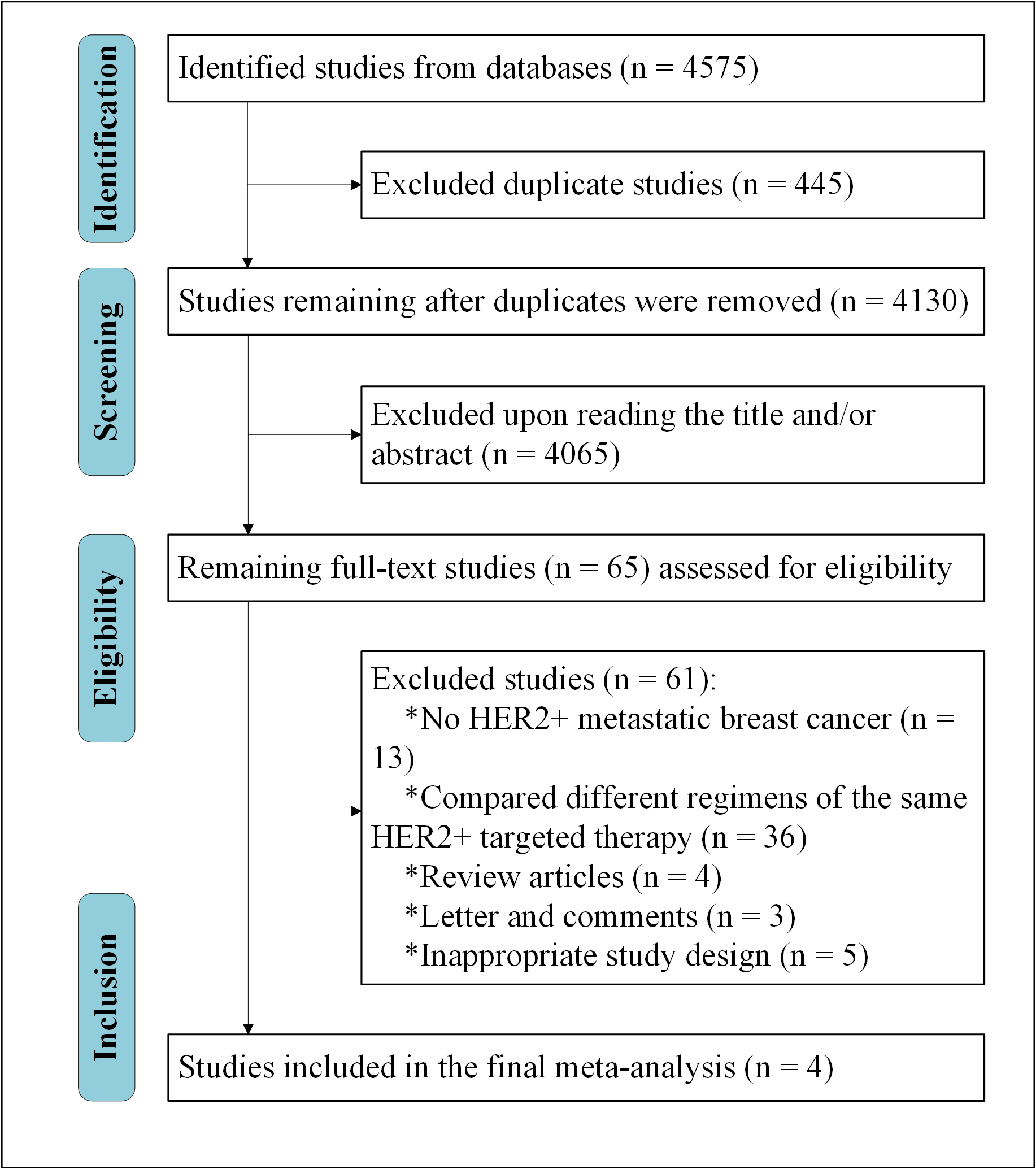


**Supplementary Fig. S2**


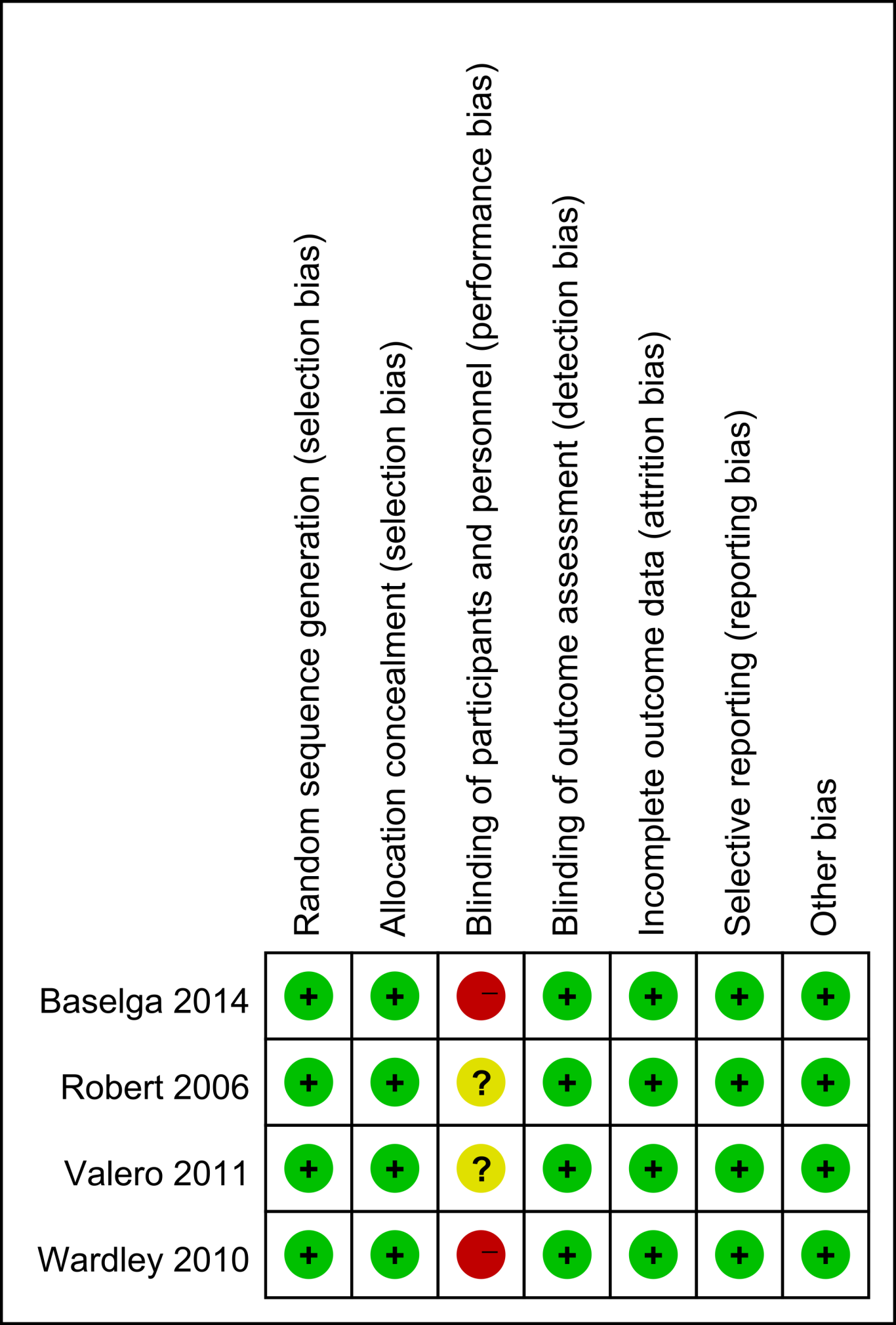


**Supplementary Fig. S3**


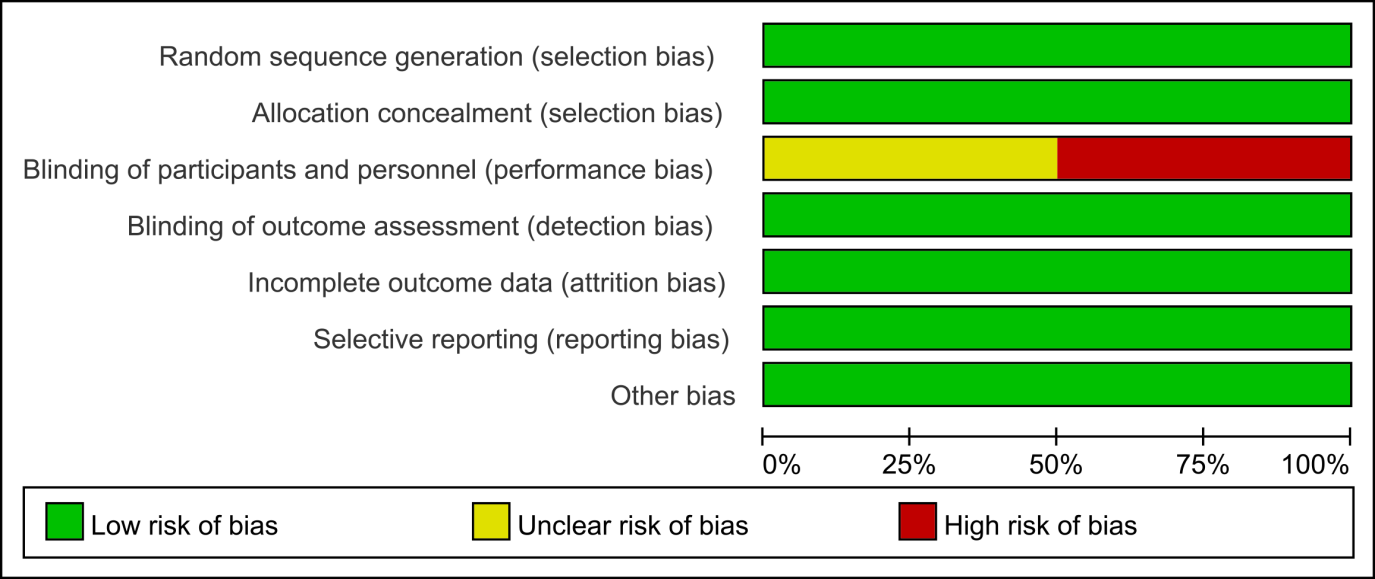


**Supplementary Fig. S4**


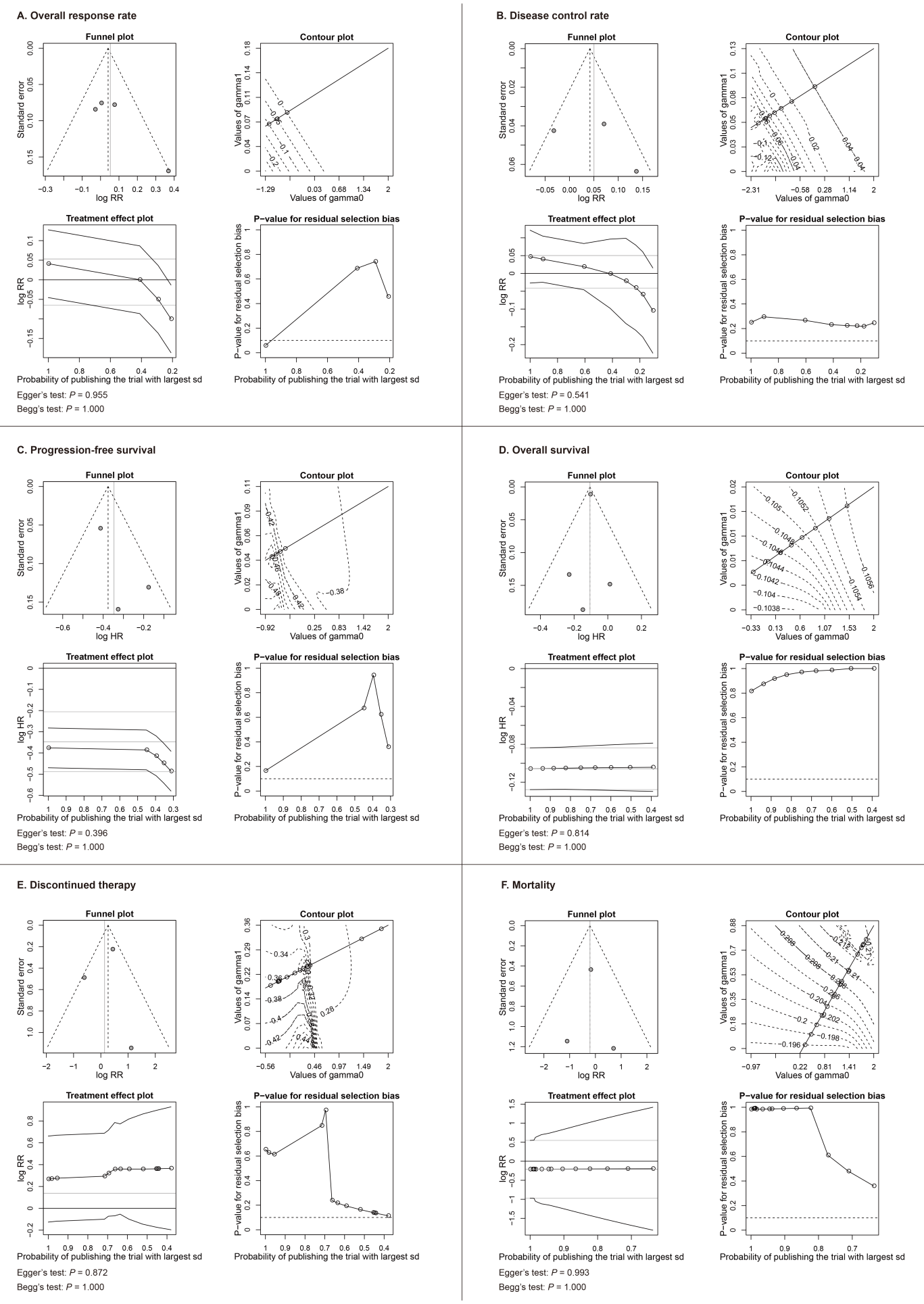


**Supplementary Fig. S5**


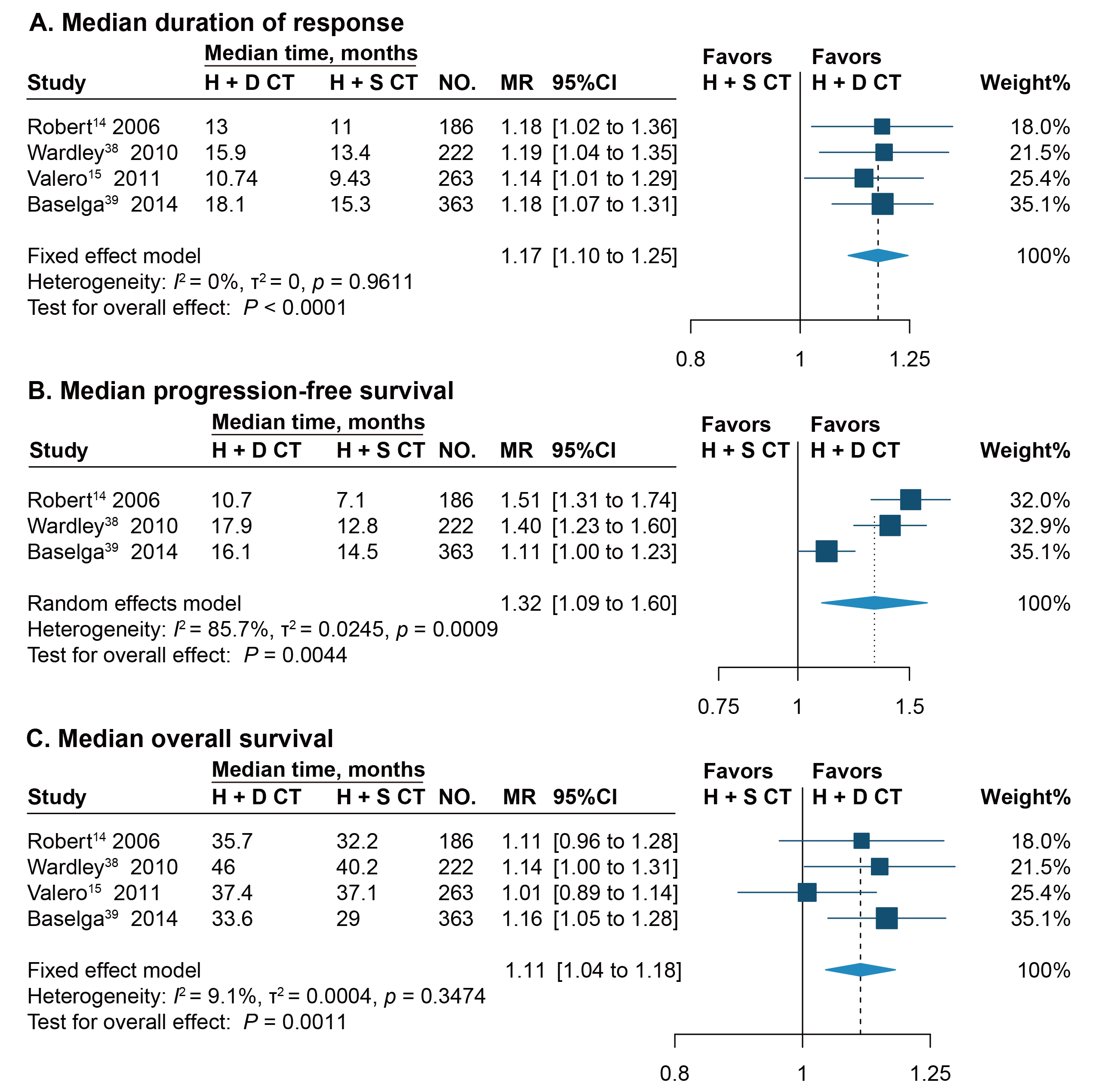


**Supplementary Fig. S6**


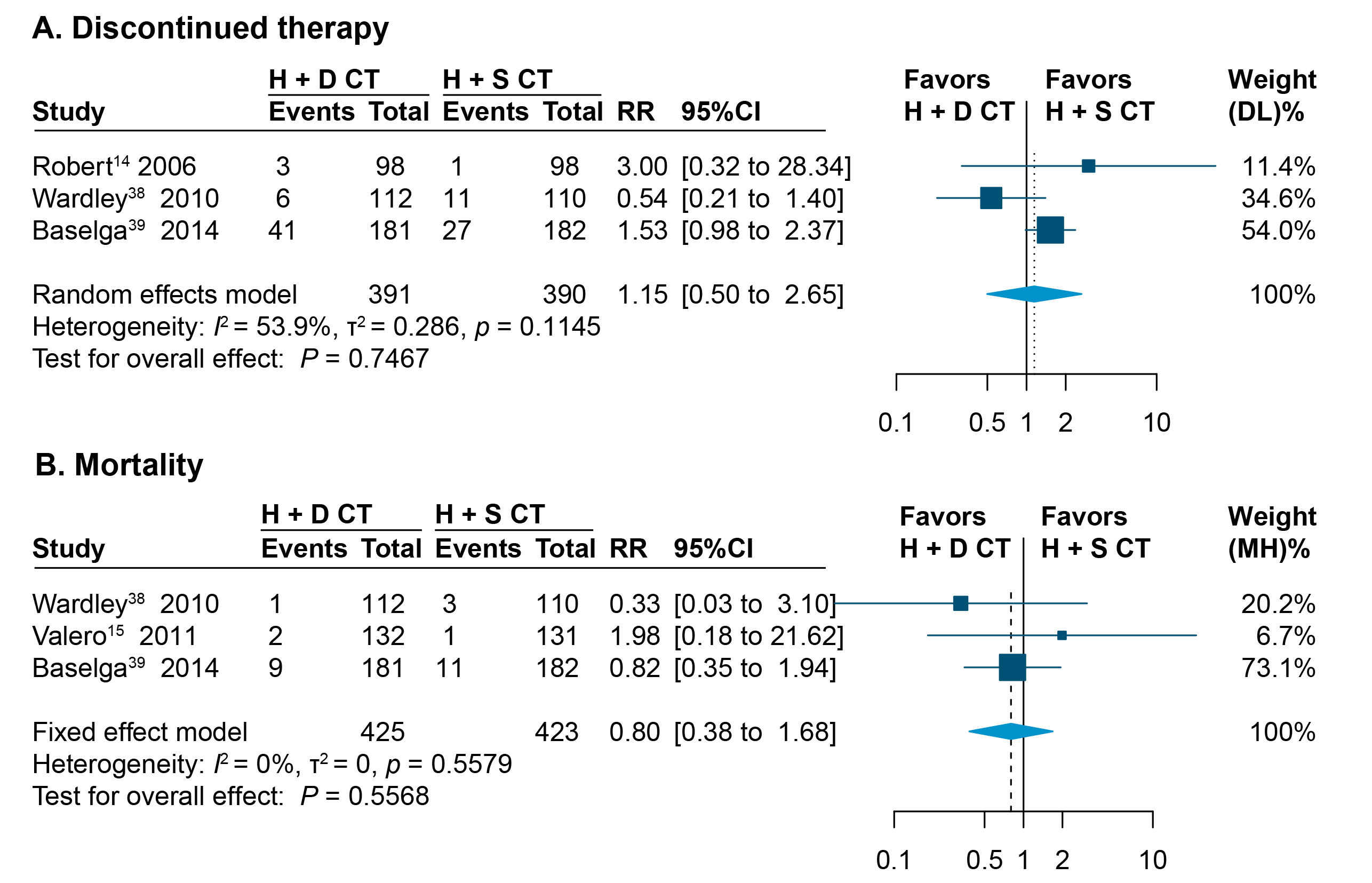


**Supplementary Tables**

**Supplementary** **Table S1.** Treatment summary for the two treatment groups.

| Study/trial | Treatment | |
| --- | --- | --- |
|  | Doublet chemotherapy + trastuzumab | Single-agent chemotherapy + trastuzumab |
| Robert et al. [14] | Trastuzumab (4 mg/kg loading dose, followed by 2 mg/kg weekly) + paclitaxel (175 mg/m^2^ q3w for 6 cycles) + Carboplatin (AUC = 6 q3w for 6 cycles) until PD | Trastuzumab (4 mg/kg loading dose, followed by 2 mg/kg weekly) + paclitaxel (175 mg/m^2^ q3w for 6 cycles) until PD |
| Wardley et al. [24] /  CHAT study /  NCT01038466 | Trastuzumab (8 mg/kg loading dose, followed by 6 mg/kg q3w) + docetaxel (75 mg/m^2^, q3w) + capecitabine (950 mg/m^2^ b.i.d, on days 1-14, q3w) until PD | Trastuzumab (8 mg/kg loading dose, followed by 6 mg/kg q3w) + docetaxel (100 mg/m^2^, q3w) until PD |
| Valero et al. [15] /  BCIRG 007 /  NCT00047255 | Trastuzumab (4 mg/kg loading dose, followed by 2 mg/kg weekly on days 1, 8, and 15 every 3 weeks for eight cycles, followed by 6 mg/kg q3w) + docetaxel (75 mg/m^2^ q3w for 8 cycles) + carboplatin (AUC = 6 q3w for 8 cycles) until PD | Trastuzumab (4 mg/kg loading dose, followed by 2 mg/kg weekly on days 1, 8, and 15 every 3 weeks for eight cycles, followed by 6 mg/kg q3w) + docetaxel (100 mg/m^2^ q3w for 8 cycles) until PD |
| Baselga et al. [25] /  NCT00294996 | Trastuzumab (4 mg/kg loading dose, followed by 2 mg/kg weekly) + paclitaxel (80 mg/m^2^ weekly for 6 cycles) + nonpegylated-liposomal-doxorubicin (50 mg/m^2^ q3w for 6 cycles) until PD | Trastuzumab (4 mg/kg loading dose, followed by 2 mg/kg weekly) + paclitaxel (80 mg/m^2^ weekly for 6 cycles) until PD |

Abbreviations: AUC, area under the curve; PD, progression of disease.

**Supplementary Table S2. Summary of the safety results for the two treatment regimens.**

| Outcomes | No. of | Relative effect | | | Risk difference | | GRADE | | | |
| --- | --- | --- | --- | --- | --- | --- | --- | --- | --- | --- |
|  | participants | Risk ratio (95% CI) | *P* value | *I*^2^ | (95% CI) | | Quality | | Importance | |
| Grade 3 or 4 hematologic toxic effects | | | | | | | | |  | |
| Neutropenia | 775 [14,24,25] | 2.38 (0.45 to 12.65) | 0.311 | 98.6% | 25% (-32% to 83%) | | ⊕OOO   Very low ^a,b,c^ | | Important | |
| Leukopenia | 580 [24,25] | 2.79 (0.09 to 88.76) | 0.561 | 98.8% | 15% (-57% to 88%) | | ⊕OOO   Very low ^a,b,c^ | | Important | |
| Thrombocytopenia | 1024 [14,15,24,25] | 4.08 (2.00 to 8.35) | 0.000 | 40.4% | 5% (3% to 8%) | | ⊕⊕⊕O   Moderate ^a^ | | Important | |
| Anemia | 685 [14,15,24] | 1.87 (0.92 to 3.80) | 0.082 | 0.0% | 3% (-0.3% to 6%) | | ⊕⊕OO  Low ^a,b^ | | Important | |
| Febrile neutropenia | 817 [14,15,25] | 2.58 (0.38 to 17.61) | 0.335 | 84.9% | 3% (-5% to 11%) | | ⊕OOO   Very low ^a,b,c^ | | Important | |
| Hemoglobin | 360 [25] | 2.02 (0.89 to 4.61) | 0.094 | - | 5% (-1% to 10%) | | ⊕⊕OO  Low ^a,b^ | | Important | |
| Grade 3 or 4 non-hematologic toxic effects | | | | | | | | |  | |
| Fatigue | 1039 [14,15,24,25] | 1.72 (0.98 to 3.02) | 0.060 | 0.0% | 3% (-0% to 5%) | | ⊕⊕OO  Low ^a,b^ | | Important | |
| Asthenia | 844 [14,15,25] | 1.55 (0.96 to 2.51) | 0.073 | 0.0% | 3% (-0.2% to 7%) | | ⊕⊕OO  Low ^a,b^ | | Important | |
| Neuropathy | 457 [14,15] | 0.92 (0.43 to 1.94) | 0.818 | 44.2% | -1% (-5% to 4%) | | ⊕⊕OO  Low ^a,b^ | | Important | |
| Nausea/vomiting | 1039 [14,15,24,25] | 4.26 (1.70 to 10.69) | 0.002 | | 0.0% | 4% (2% to 5%) | | ⊕⊕⊕O  Moderate ^a^ | | Important |
| Diarrhea | 1038 [14,15,24,25] | 2.81 (1.48 to 5.37) | 0.002 | | 0.0% | 4% (2% to 7%) | | ⊕⊕⊕O  Moderate ^a^ | | Important |
| Anorexia | 777 [14,24,25] | 3.00 (0.82 to 10.96) | 0.098 | | 16.2% | 2% (-0.2% to 3%) | | ⊕⊕OO  Low ^a,b^ | | Important |
| Fever | 777 [14,24,25] | 1.66 (0.52 to 5.30) | 0.391 | | 32.8% | 1% (-1% to 3%) | | ⊕⊕OO  Low ^a,b^ | | Important |
| Stomatitis | 844 [14,15,25] | 5.02 (1.73 to 14.55) | 0.003 | | 38.1% | 4% (2% to 6%) | | ⊕⊕⊕O  Moderate ^a^ | | Important |
| Dyspnea | 555 [14,25] | 0.86 (0.29 to 2.52) | 0.783 | | 0.0% | -0.4% (-3% to 2%) | | ⊕⊕OO  Low ^a,b^ | | Important |
| Congestive heart failure | 459 [14,24] | 0.25 (0.03 to 2.21) | 0.211 | | 0.0% | -1% (-3% to 1%) | | ⊕⊕OO  Low ^a,b^ | | Important |
| Nail disorder | 582 [24,25] | 0.75 (0.07 to 8.17) | 0.812 | | 68.0% | -1% (-6% to 4%) | | ⊕OOO  Very low ^a,b,c^ | | Important |
| Peripheral edema | 844 [15,24,25] | 0.36 (0.13 to 0.98) | 0.046 | | 0.0% | -2% (-4% to -0.1%) | | ⊕⊕⊕O  Moderate ^a^ | | Important |
| Arthralgia | 484 [15,24] | 0.60 (0.08 to 4.46) | 0.614 | | 0.0% | -0.4% (-2% to 1%) | | ⊕⊕OO  Low ^a,b^ | | Important |
| Myalgia | 484 [15,25] | 0.17 (0.02 to 1.37) | 0.095 | | 0.0% | -2% (-4% to 0%) | | ⊕⊕OO  Low ^a,b^ | | Important |
| Allergic reaction | 195 [14] | 1.98 (0.37 to 10.56) | 0.424 | | | - | 2% (-3% to 7%) | | ⊕⊕OO  Low ^a,b^ | Important |
| Pain | 195 [14] | 0.72 (0.30 to 1.71) | 0.457 | | | - | -3% (-12% to 5%) | | ⊕⊕OO  Low ^a,b^ | Important |
| Infection | 195 [14] | 0.99 (0.14 to 6.89) | 0.992 | | | - | 0% (-4% to 4%) | | ⊕⊕OO  Low ^a,b^ | Important |
| Hypomagnesemia | 195 [14] | 4.95 (0.24 to 101.78) | 0.300 | | | - | 2% (-1% to 6%) | | ⊕⊕OO  Low ^a,b^ | Important |
| Alopecia | 222 [24] | 0.76 (0.30 to 1.98) | 0.579 | | | - | -1% (-9% to 5%) | | ⊕⊕OO  Low ^a,b^ | Important |
| Hand-foot syndrome | 222 [24] | 22.27 (2.92 to 169.53) | 0.004 | | | - | 16% (9% to 23%) | | ⊕⊕OO  Low ^a,b^ | Important |
| Mucosal inflammation | 222 [24] | 0.49 (0.05 to 5.34) | 0.559 | | | - | -1% (-4% to 2%) | | ⊕⊕OO  Low ^a,b^ | Important |
| Constipation | 222 [24] | 2.95 (0.12 to 71.57) | 0.507 | | | - | 1% (-2% to 3%) | | ⊕⊕OO  Low ^a,b^ | Important |
| Rash/desquamation | 262 [15] | 0.33 (0.04 to 3.16) | 0.339 | | | - | -2% (-5% to 1%) | | ⊕⊕OO  Low ^a,b^ | Important |
| Alanine aminotransferase | 360 [25] | 2.43 (0.87 to 6.75) | 0.089 | | | - | 4% (-0.4% to 8%) | | ⊕⊕OO  Low ^a,b^ | Important |
| Alkaline phosphatase | 360 [25] | 1.42 (0.65 to 3.10) | 0.385 | | | - | 2% (-3% to 7%) | | ⊕⊕OO  Low ^a,b^ | Important |
| Aspartate  aminotransferase | 360 [25] | 0.58 (0.17 to 1.94) | | 0.375 | | - | | -2% (-5% to 2%) | ⊕⊕OO  Low ^a,b^ | Important |
| Peripheral neuropathy | 360 [25] | 1.01 (0.48 to 2.12) | | 0.977 | | - | | 0.1% (-5% to 5%) | ⊕⊕OO  Low ^a,b^ | Important |
| Peripheral sensory  neuropathy | 360 [25] | 1.17 (0.57 to 2.38) | | 0.672 | | - | | 1% (-4% to 7%) | ⊕⊕OO  Low ^a,b^ | Important |
| Hypertension | 360 [25] | 1.30 (0.50 to 3.42) | | 0.594 | | - | | 1% (-3% to 5%) | ⊕⊕OO  Low ^a,b^ | Important |
| Erythema | 360 [25] | 1.35 (0.31 to 5.94) | | 0.693 | | - | | 0.6% (-2% to 3%) | ⊕⊕OO  Low ^a,b^ | Important |
| Paresthesia | 360 [25] | 0.40 (0.08 to 2.06) | | 0.275 | | - | | -1% (-5% to 1%) | ⊕⊕OO  Low ^a,b^ | Important |
| Dry skin | 360 [25] | 2.53 (0.50 to 12.86) | | 0.264 | | - | | 2% (-1% to 5%) | ⊕⊕OO  Low ^a,b^ | Important |
| Onycholysis | 360 [25] | 2.02 (0.51 to 7.96) | | 0.314 | | - | | 2% (-2% to 5%) | ⊕⊕OO  Low ^a,b^ | Important |
| Study withdrawals |  |  | |  | |  | |  |  |  |
| Discontinued therapy * | 781 [14,24,25] | 1.15 (0.50 to 2.65) | | 0.747 | | 53.9% | | 2% (-5% to 8%) | ⊕OOO   Very low ^a,b,c^ | Important |
| Mortality ** | 848 [15,24,25] | 0.80 (0.38 to 1.68) | | 0.557 | | 0.0% | | -1% (-3% to 2%) | ⊕⊕OO  Low ^a,b^ | Important |

Abbreviations: CI, confidence interval; CT, chemotherapy.

* Patient discontinued therapy due to treatment-related toxicities.

** Patient mortality during the period of drug therapy.

GRADE Working Group grades of evidence:

⊕⊕⊕⊕ **High quality:** Further research is very unlikely to change our confidence in the estimated effect.

⊕⊕⊕O **Moderate quality:** Further research is likely to have an important impact on our confidence in the estimated effect and might change the estimate.

⊕⊕OO **Low quality:** Further research is very likely to have an important impact on our confidence in the estimated effect and might change the estimate.

⊕OOO **Very low quality:** We are very uncertain regarding the estimate.

^a^ Downgraded (-1) for risk of bias: All trials were judged as having an unclear or high risk of bias related to the blinding of participants and personnel.

^b^ Downgraded (-1) for imprecision: Small sample bias may exist, or 95% confidence intervals with respect to the absolute effects are wide.

^c^ Downgraded (-1) for inconsistency: Substantial heterogeneity (*I*^2^ > 50%) was identified among the trials.
